# Liposarcoma: A ‘Beer Belly’ in Disguise

**DOI:** 10.7759/cureus.28067

**Published:** 2022-08-16

**Authors:** Nicholas D Luke, Alexander Gart, Raji Mohammad, Ali Raza

**Affiliations:** 1 School of Medicine, St. George's University School of Medicine, True Blue, GRD; 2 Department of Surgery, New York City Health and Hospitals Corporation (NYCHHC), Bronx, USA; 3 Department of Pathology, New York City Health and Hospitals Corporation (NYCHHC), Bronx, USA; 4 Department of Surgical Oncology, New York City Health and Hospitals Corporation (NYCHHC), Bronx, USA

**Keywords:** mdm2, retroperitoneum, ureter compression, surgical oncology, well differentiated liposarcoma

## Abstract

Liposarcoma is a locally aggressive tumor that may originate in soft tissue sites such as the retroperitoneum or the extremities, or less frequently, from the bone. The fatty tumor may have an insidious growth pattern and be present incidentally on imaging, or it may be present with symptoms such as small bowel or ureter obstruction. The diagnosis can be confirmed post-operatively via fluorescence in situ hybridization (FISH) with the presence of mouse double minute 2 (MDM2) homolog protein and cyclin-dependent kinase 4 (CDK4) gene amplification. The rate of recurrence may be high depending on the subtype of liposarcoma, so it is always recommended to have the patient undergo routine imaging every six months to a year. In this case report, we present a patient who presented with a massive, incidental liposarcoma found on imaging after coming to the emergency department for lower extremity trauma.

## Introduction

Liposarcoma is a rare mesenchymal neoplasm that originates within the deep adipose tissue of the body. It is a malignant tumor that can grow anywhere in the body and cause a mass effect if it gets too large. The most commonly involved sites are the esophagus, retroperitoneum, and the popliteal fossa [1]. Although the exact etiology of liposarcoma is not fully understood, the American Cancer Society has identified potential risk factors, including radiation exposure/therapy, trauma to the lymphatic system, toxic chemical exposure, and having a family history of cancer-prone diseases [1]. Lee et al. describe how two specific subtypes of liposarcoma, well-differentiated and dedifferentiated, have very different properties regarding metastasis, prognosis, and treatment response [2]. However, both subtypes have common recurrent gene amplifications on chromosome 12 that lead to adipocytic malignant transformation and are possible therapeutic targets [2]. According to Yang et al., liposarcoma generally has not been sensitive to radiotherapy or chemotherapy in the past and has had a poor prognosis. However, advances in modern science via genomic technology have made treating and managing liposarcoma far more successful [3]. In this case report, we present a patient who had a massive occult liposarcoma develop in his peritoneum for an unknown number of years only to be discovered incidentally during an emergency department visit for an unrelated complaint.

## Case presentation

Our presenting patient is a 49-year-old male with no past medical history who presented to the emergency department with a tier two activation status post moped versus motor vehicle event. The patient was an unhelmeted moped rider going approximately 25 miles per hour when he was struck on the left side, throwing the patient off the moped. The patient denies any head injury, loss of consciousness, or neck pain. The patient had stable vital signs and a Glasgow coma scale score of 15 upon arrival at the emergency department. Upon evaluation, the patient denied trauma-related complaints. A review of systems was positive for myalgia in the left lower extremity. The patient had no past surgical or medical history, no known allergies, and no family history of morbidities or cancer.

The primary survey of the patient showed an intact airway, no distress, no shock symptoms, a Glasgow coma scale score of 15 (eyes open spontaneously, oriented, and obey commands), and trauma to the left ankle. His vital signs on admission were: a temperature of 98.7 °F (37.1 °C), a heart rate of 79 beats per minute, a respiratory rate of 23 breaths per minute, an oxygen saturation of 96%, and a blood pressure of 134/72 mmHg. The patient was normal and in no distress, and the physical exam was remarkable for tenderness and swelling present over the left ankle along the lateral malleolus with decreased range of motion. The abdominal exam demonstrated a soft protuberant abdomen without a fluid wave and with no discrete mass. A complete blood count was remarkable for an elevated white blood cell count (13.08 × 10^3^/mcL), consisting of 75.2% neutrophils and 15.7% lymphocytes, and an absolute neutrophil count of 9.82 × 10^3^/mcL. An arterial blood gas analysis resulted in a pH of 7.362, a PCO_2_ of 54.5 mmHg, and an HCO_3_ of 30.9 nmol/L.

**Figure 1:**
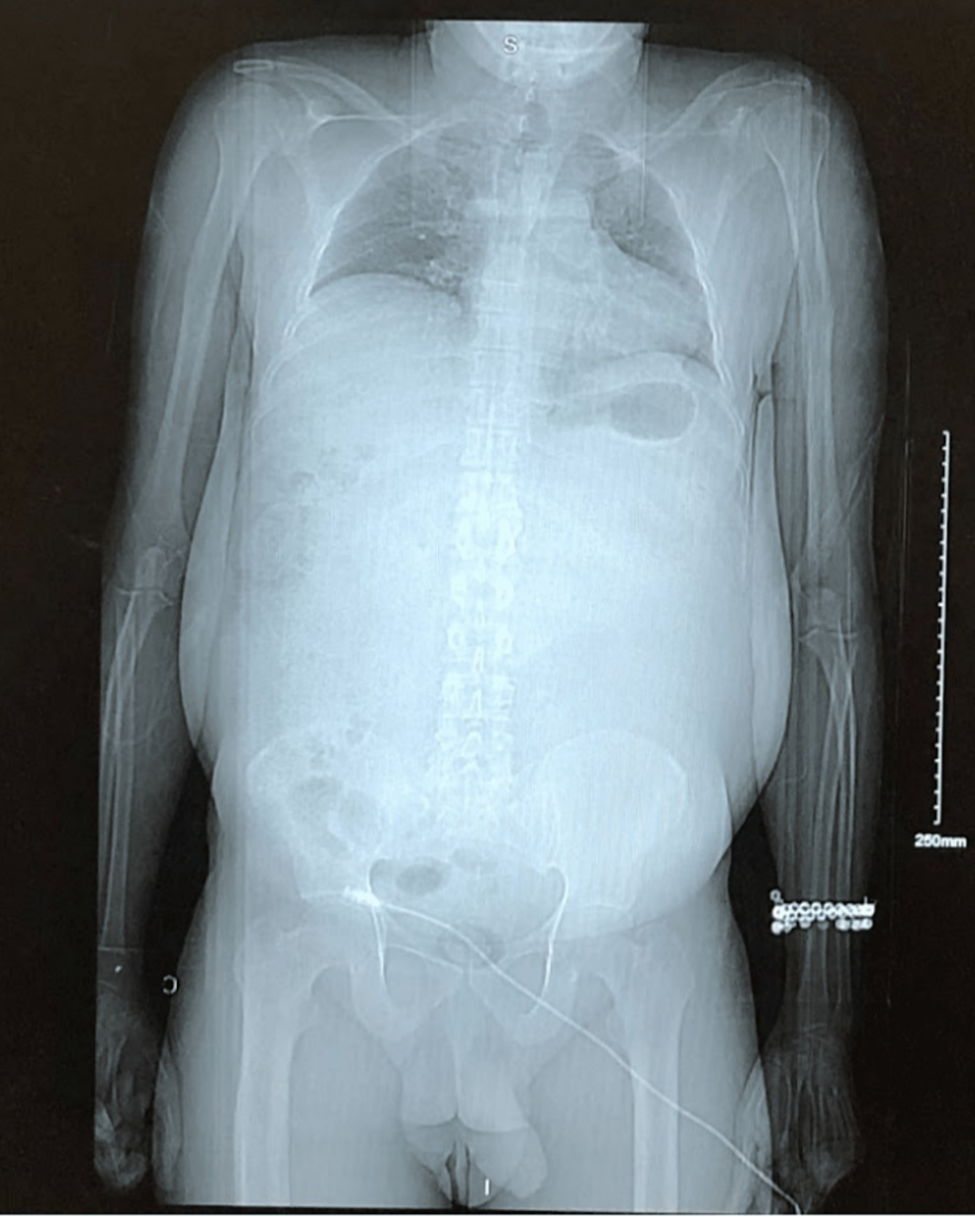
Scout computed tomography of the abdomen without contrast. The patient had a large abdomen relative to the body habitus.

**Figure 2:**
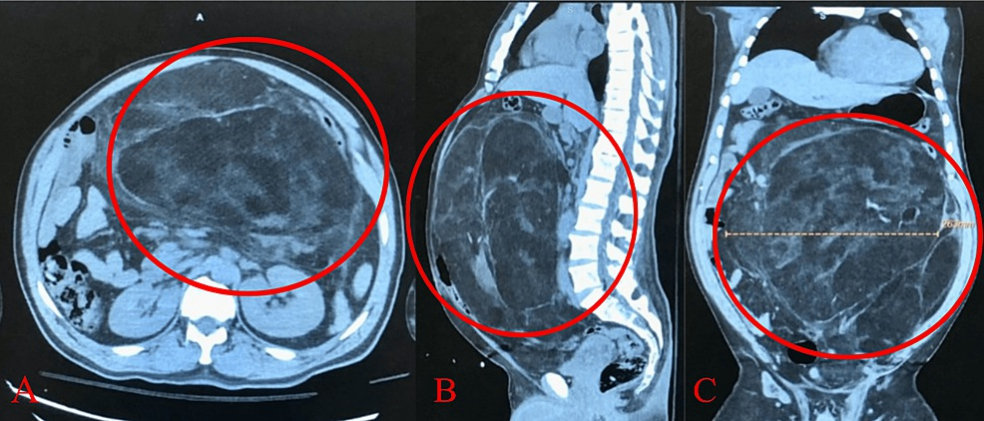
Initial computed tomography imaging without contrast in the emergency department results showed a 32 cm incidental lipomatous lesion of the left hemiabdomen with mass effect on the bowel and with mild soft tissue compartment in the axial (red circle in A), sagittal (red circle in B), and coronal planes (red circle in C).

**Figure 3:**
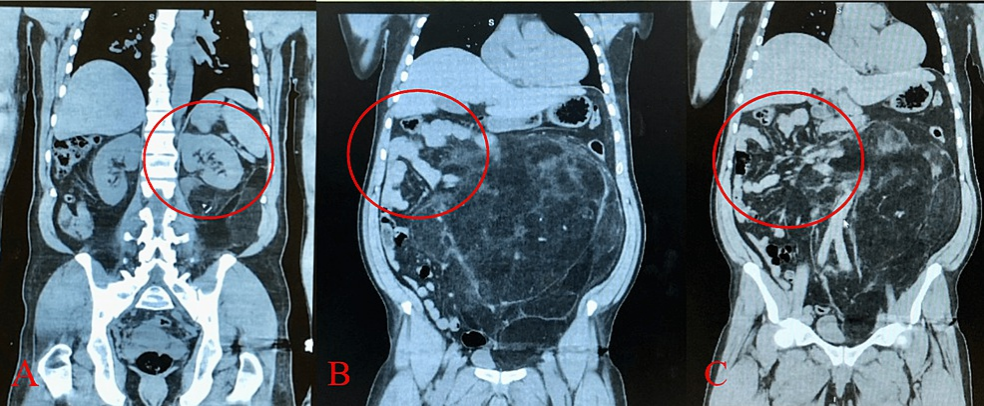
Initial coronal computed tomography without contrast imaging depicted a left kidney displaced transversely due to the lipomatous mass (red circle in A). Not shown: the left ureter was largely deviated to the right hemiabdomen at its upper portion due to the mass and returned to the left hemiabdomen at the renal pelvis. Other computed tomography image findings depicted the mass pushing the left colon into the right hemiabdomen (red circles in B, C). All organ structures involved (left kidney, left ureter, colon, aorta) were viable throughout the patient’s hospital stay.

**Figure 4:**
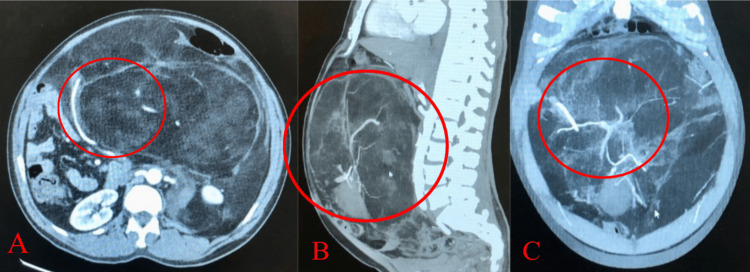
Computed tomography angiography with intravenous contrast depicted a large lipomatous abdominal mass extending from the upper abdomen to the pelvis and measured approximately 32 cm, in the axial (A), sagittal (B), and coronal planes (C). Internal septations were noted. Arterial supply appeared to be originating from the inferior mesenteric artery and a secondary branch was noted on the infrarenal abdominal aorta (red circles). Venous drainage appeared to be via the inferior mesenteric vein.

An initial computed tomography scan without contrast study of the abdomen and pelvis was ordered in order to rule out occult trauma and fractures, and the results showed a 32-cm incidental lipomatous lesion of the left hemiabdomen with a mass effect on the bowel and a mild soft tissue compartment (Figures 1, 2A-2C, 3A-3C). The patient was further interviewed about the finding, and he relayed increased abdominal girth for over 1.5 years that he attributed to ‘a beer belly'. The patient stated that he was a social drinker (a few beers on the weekend), and said that he never had any symptoms in the past related to the mass effect of the large fatty mass (such as constipation or flank/abdominal pain). This finding prompted a follow-up of the mass with computed tomography with intravenous contrast of the abdomen and pelvis. The result showed a large lipomatous abdominal mass extending from the upper abdomen to the pelvis and measuring approximately 32 cm in the craniocaudal dimension (Figure 4A-4C). Internal septations were seen. Arterial supply appeared to be originating from the inferior mesenteric artery and a secondary branch was noted on the infrarenal abdominal aorta. Venous drainage appeared to be via the inferior mesenteric vein. There was no significant retroperitoneal, pelvic side, or inguinal lymphadenopathy. There was a significant displacement of the small bowel and colon, predominantly to the right, and there was no pneumoperitoneum or free fluid within the peritoneal cavity or pelvis.

The tumor board agreed to resect the mass with an R0/R1 margin. An abdominal exploration with en bloc resection, bilateral ureteral stents, and a central line was planned, with possible bowel resection and ostomy. The patient was taken to the operating room and underwent general anesthesia. Under fluoroscopy, Urology inserted bilateral ureteral stents. The left ureter was largely deviated to the right hemiabdomen at its upper portion due to the mass and returned to the left hemiabdomen at the renal pelvis. The stents were secured and affixed to foley bags. Under sterile conditions, a central line was placed in the right jugular vein for the possibility of a large volume of blood loss.

Next, the abdomen was prepped, and the sterile field extended from the nipples down to the femoral vessels. A midline incision extending from the xiphoid to the pubis was made. The midline of the fascia was encountered and was extremely attenuated and thin without any hernias. A well-circumscribed multilobulated fatty golden lesion with engorged veins along its capsule was immediately encountered. The mass appeared soft and partially mobile. The left colon had been lifted up by this mass and pushed over to the right hemiabdomen across the midline. The mobilization of the left colon was carried off by the right lateral extent of the mass. The colonic mesentery was seen clearly and was not violated; it was fixed to the capsule of the mass over an approximately 15 cm portion and was reselected; the left colonic artery and its arcades were not violated. The blood supply of the colon was assessed throughout the case and remained intact, with the left colon remaining stable. The superior portion of the mass abutted the transverse colon mesentery; however, this plane was similarly adherent without invasion. The dissection began at the left paracolic gutter, and the mass was rotated towards the patient’s lateral left retroperitoneal fat and sigmoid colon, which were uninvolved.

The small bowel was entirely displaced to the right hemiabdomen and remained viable throughout the entire case, as did the colon too. The overall dimensions of the mass were approximately 40 cm × 40 cm and extended to the paracolic gutters, inferiorly to the pelvis, and posteriorly toward the aorta and retroperitoneum. The next resection occurred towards the pelvis on the left side wall. A dense adhesion was noted in the left inguinal region where the mass was affixed to the prior unknown inguinal hernia repair mesh. The mesh was taken as part of the specimen and no gross tumor remained. Afterward, the posterolateral surface of the mass was mobilized away from the left ureter and the ureter vessels. The ureter abutted the mass and was freed without the need for resection. Gerota’s fascia was taken as part of the specimen.

It was noted that a poor plane between the more medial aorta and the fatty mass was present, so a meticulous serial dissection of the retroperitoneal fat was taken along with the capsule of the mass, skeletonizing the aorta and adjacent psoas muscle.

Deep dissection of the mass was achieved by mobilization of the aorta and left iliac vessels. A small amount of bleeding was encountered from a branch of the internal iliac artery and this was controlled using a 6-0 Prolene suture (Ethicon Store, USA). The most inferior portion of the mass was mobilized off of the external iliac artery and the vein and small venous branches were ligated using clips and the LigaSure device (Covidien, Ireland).

**Figure 5:**
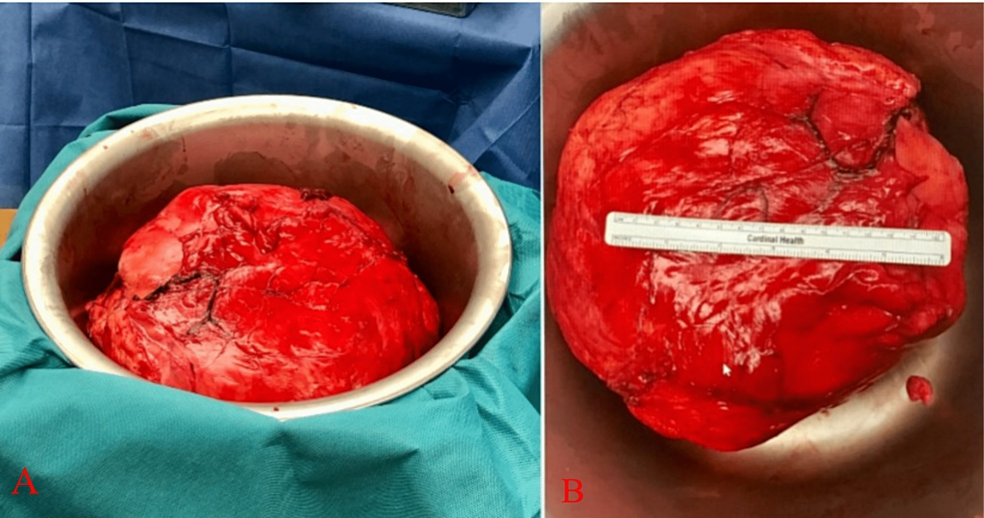
Gross images of the well-circumscribed, soft, fatty, 30 cm × 32 cm × 15 cm mass obtained intraoperatively. The total weight was 8150 grams.

At this point, the entire mass was free and handed off the field (Figure 5A-5B). The ureter, bladder, aorta, small bowel, and left colon were inspected and no damage was seen, and all exhibited strong pulses. Raw oozing in the retroperitoneum was controlled with electrocautery. The dissected plane was irrigated with three liters of warm saline, and the returning effluent was clear. The bowels exhibited strong peristalsis, and any oozing sites were cauterized and observed for hemostasis. The mesenteric defect in the left colon was closed using a 3-0 Vicryl suture (Ethicon Store, USA), and the arcade of Riola and Drummond was entirely visible throughout. The patient's abdomen was scaphoid after the removal of the mass. The fascia was then closed using two separate #1 looped polydioxanone (PDS) sutures placed in a running fashion and tied to one another. Seprafilm (Sanofi, France) was placed in the midline prior to closure and the omentum swept. Estimated blood loss was about 250 ml, urine output was about 700 ml, and 7 l of fluid was administered.

Stents were removed on postoperative day 2 with no complications. On postoperative day 4, two units of blood were given. The patient was started on a nil per os (NPO), or nothing by mouth, diet with intravenous fluids for seven days and then was placed on a general diet.

**Figure 6:**
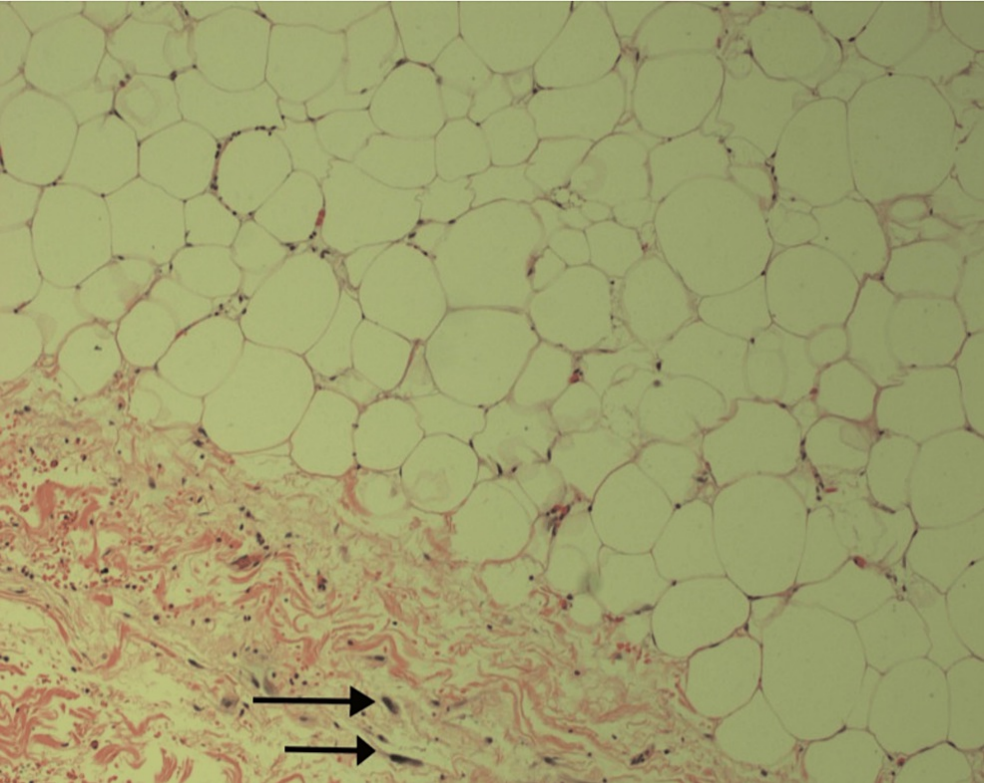
Histological imaging depicted that liposarcoma is made of well-differentiated adipose tissue with scattered atypical cells with hyperchromatic nuclei (black arrows). Original magnification was ×200, histological stain used was hematoxylin and eosin.

**Figure 7:**
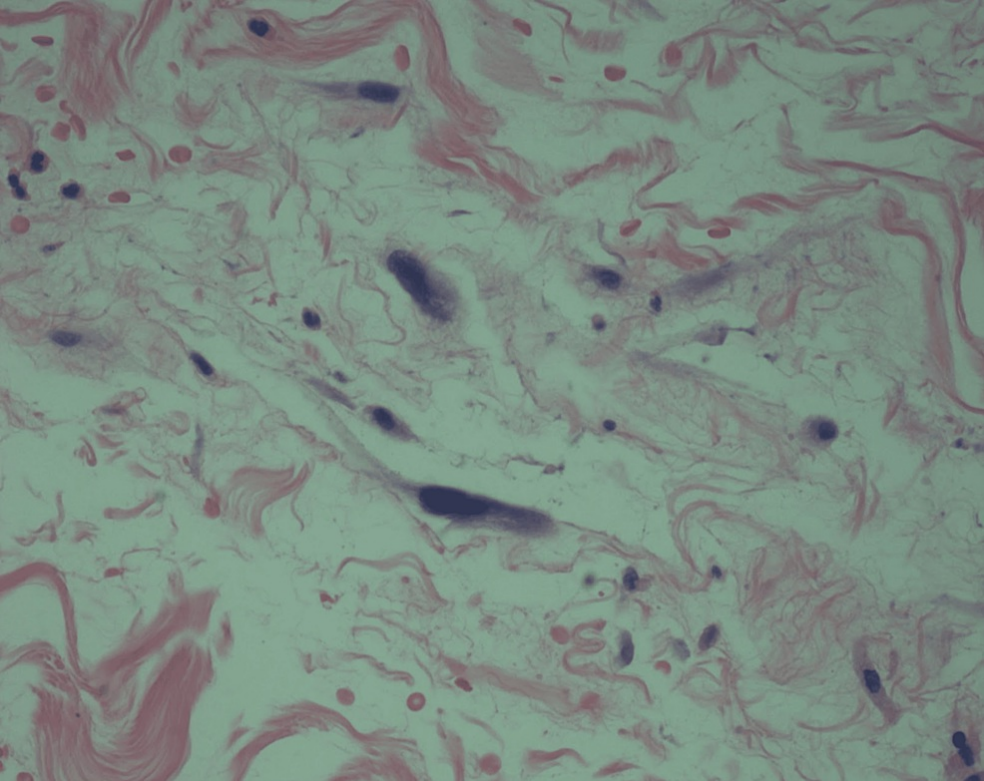
High magnification of histologic image from previous figure depicting atypical cells (×400 magnification, with hematoxylin and eosin stain).

The surgical pathology report confirmed that the mass was a well-differentiated liposarcoma that was mouse double minute 2 (MDM2) positive/amplified on fluorescence in situ hybridization (FISH). The results of FISH included an MDM2/CEP 12 ratio of 4.8:1, an MDM2 mean copy number of 9.7, a chromosome numeration probe 12 (CEP 12) mean copy number of 2.0, and 50 tumor cells were scored. Histology showed well-differentiated adipose tissue with scattered atypical cells with hyperchromatic nuclei on hematoxylin and eosin stain (Figures 6-7). The mass weighed about 8150 g.

## Discussion

Well-differentiated liposarcoma is a painless tumor and is the most common subtype of liposarcoma. The tumor does not display a gender prevalence and typically occurs in adults over the age of 50 at a rate of 2 cases for every 1,000,000 people [4]. The most common locations of liposarcomas include the retroperitoneum and proximal extremities such as the thigh [4]. Well-differentiated liposarcomas are typically locally aggressive, do not metastasize, and can grow very large and go unnoticed since they do not produce pain [4]. Just like in our presenting patient, well-differentiated liposarcomas may develop a capsule and become multilobular as they grow. The prognosis is based on the location and margins of the tumor. A well-differentiated liposarcoma on the extremities has shown 95% disease-specific survival, whereas the retroperitoneum had an 87% disease-specific survival rate [4].

A standard approach for treating liposarcoma involves surgical resection. While this method is effective in removing the tumor and clearing its margins, recurrence is highly probable. The recurrence rate depends on size and location; 13-46% for well-differentiated liposarcoma in the extremities and 91% for retroperitoneal cases [4]. In order to combat this, it is more viable to use a multimodality approach to apply long-term treatment for liposarcoma. Although radiation and chemotherapy have low response rates in well-differentiated liposarcoma, the use of novel agents targeting chromosome 12 gene products MDM2 and cyclin-dependent kinase 4 (CDK4) has shown promise in pre-clinical studies and is being tested in clinical trials [5]. Other tools for genomic analysis, such as cell line examinations and tissue microarray, have identified additional targets including zinc finger of the cerebellum 1 (ZIC1), topoisomerase II-alpha (TOP2A), aurora A kinase (AURKA), and insulin-like growth factor 1 receptor (IGF-1R) for therapeutic use [5]. Crago and Singer describe how retrospective data were taken on patients with all types of liposarcomas and how the surgeons used a "frontline aggressive approach that entails routine resection of adjacent viscera" [5]. This was done in order to provide even more normal tissue around the tumor, and the data showed that the local recurrence rate at 5 years (22%) was lower than in historical controls (41%), but at the cost of increased surgical morbidity [5].

One thing to note about our presenting patient is how the resection of the mass was performed. Along with a lack of sufficient exposure, the large lipomatous mass had to be segmented and removed piece by piece. Ideally, the mass should have been removed as one whole piece with the capsule, but the capsule also had to be broken in order to remove the tumor from its infrarenal origin. In one example, Hashimoto et al. described a case in which a liposarcoma was successfully resected in one piece with no signs of rupture [6]. The patient described by Hashimoto et al. had no complications or tumor recurrences postoperatively in the first nine months, and the resected tumor margins were negative microscopically [6]. While our presenting patient had no acute postoperative complications, we should continue to observe our patient in an outpatient setting so that we may ensure that the tumor margins remain negative.

If the patient had not presented to the emergency room for his left ankle trauma, the liposarcoma would not have been discovered. This incidental finding could potentially have saved the patient from further comorbidities such as small bowel obstruction and acute kidney injury. If signs of the latter had occurred, it would be possible to see metastasis of the liposarcoma as it would have been given enough time to grow insidiously and seed distant organs via hematogenous dissemination. Duman et al. presented a case in which a liposarcoma had grown large enough to cause an acute kidney injury [7]. In their presenting patient, both ureters had been compressed, which caused elevated blood urea nitrogen (BUN) and creatinine levels [7]. According to Duman et al., the mass had grown to 4000 g when it began to exhibit mass effects within the retroperitoneum, and it could have progressed into abdominal compartment syndrome if left untreated [7]. Although our patient did not have renal symptoms or signs of abdominal compartment syndrome, the detection of left ureter compression early on prevented a future acute kidney injury and demonstrated that the tumor in our patient had begun to exhibit a mass effect.

Since our patient had a positive MDM2 result on the pathology report, theoretically, it is possible to perform genetic tests on the patient's family in order to determine their risk of liposarcoma. Focusing on amplifications of the MDM2 and CDK4 gene products may be especially helpful as these amplifications have a strong association with the occurrence of well-differentiated liposarcoma [8]. These gene amplification products may not always be specific to liposarcoma subtypes, but they can be utilized as potential diagnostic markers via FISH and quantitative polymerase chain reaction [8]. According to Alexio et al., it is not advisable to use MDM2 and CDK4 individually as diagnostic markers [9]. This is due to the low specificity of MDM2 (65%) and CDK4 having a low sensitivity (68.4%) [9]. Alexio et al. advocated the use of both MDM2 and CDK4 gene amplification products in order to clinch the diagnosis of a well-differentiated liposarcoma [9]. While we consider genetic testing for the patient’s family, we also need to discuss the possibility of recurrence of the tumor with our presenting patient. There are not many ways to prevent liposarcoma. We may educate the patient on avoiding chronic toxic chemicals and radiation exposure, however, there is still no guarantee that the patient is in the clear. Our presenting patient had a well-differentiated liposarcoma of infrarenal origin, which means that the tumor is located within the retroperitoneum. The location of the tumor matters because it may indicate that the tumor has a higher chance of reoccurrence. Routine computed tomography scans should be used in the surveillance phase once the initial mass has been removed.

The final pathology demonstrated positive microscopic margins. These positive margins abut the central small bowel and left colonic mesentery, and no nodal disease was apparent, and the patient requires ongoing surveillance. Surveillance is carried out with computed tomography imaging every three to six months for two to three years and then biannually. According to O'Donnell et al. [10], lower rates of tumor recurrence were seen with positive microscopic margins in comparison to gross margins. The status of the margins may play an important role in the prognosis of the patient because it has been associated with survival rates. Knebel et al. performed a retrospective study over 15 years that examined the survival rates in patients with liposarcomas [11]. The presence of positive margins made a difference in survival rates. According to Knebel, survival rates of patients with clear margins were 100%, 84.3%, and 67% after 1, 5, and 10 years, respectively [11]. Knebel et al. also noted that microscopic positive margins had corresponding survival rates of 97.7%, 81.9%, and 75.6%, respectively [11]. Finally, Knebel et al. also noted that if a macroscopic positive tumor was left in place, the respective survival rates were 100%, 75.0%, and 31.3% [11]. The effects of positive margins became much more apparent in the 10-year survival window, especially if the margins were macroscopically positive.

Management of soft tissue tumors based on tumor margins is a subjective area because there are no standardized guidelines to define what the width of a margin is or what makes a margin negative [12]. The width of the margin may be determined based on anatomical location and composition. A broad margin is preferred in order to minimize tumor recurrence, but a margin involving dense regular connective tissue can be crucial in preventing tumor cell infiltration [12]. It should be noted that the efficacy of the latter is not exactly known, but may be helpful to consider in a case-by-case context [12]. Generally, systemic therapy is not recommended for low-grade tumors. Management of positive margins may also include the utilization of adjuvant radiotherapy, but utilization for low-grade tumors is not well established [13]. The National Comprehensive Cancer Network recommends adjuvant radiotherapy for more aggressive soft tissue sarcoma resections with close soft tissue (<1 cm from the tumor) or positive resection margins [13]. One issue with this guideline is that it is based on a very limited number of heterogeneous studies, according to Cates and Cates [13]. While a clinician may opt-in to utilize adjuvant radiotherapy, there is no evidence that it may reduce the risk of recurrence [13,14]. In another retrospective study performed by Yami et al., the option of a postoperative radiation boost after preoperative radiation administration was examined [15]. Yami et al. concluded that the postoperative radiation boost (external beam radiotherapy) did not play a role in preventing local recurrences in low-grade sarcomas [15]. It does not seem that the additional postoperative radiation exposure outweighs the risk of long-term radiation adverse effects in order to prevent local liposarcoma recurrences.

## Conclusions

Well-differentiated liposarcomas are fatty tumors that can grow to become extremely large and cause mass effects on surrounding neuro-vasculature and organs. These tumors are locally aggressive and should be suspected in patients with a family history. Diagnosis can be made via computed tomography imaging, and FISH and histology post-operatively. If family history is non-contributory, then having a high index of suspicion is reasonable when dealing with a patient that comes in with a distended abdomen and has symptoms that can be a result of tumor compression (compressed ureter, hydronephrosis, small bowel obstruction, etc.). Once the diagnosis has been made, the patient should undergo routine monitoring via imaging in order to assess for possible recurrences after the surgical resection.
